# mRNA vaccination boosts cross-variant neutralizing antibodies elicited by SARS-CoV-2 infection

**DOI:** 10.1126/science.abg9175

**Published:** 2021-03-25

**Authors:** Leonidas Stamatatos, Julie Czartoski, Yu-Hsin Wan, Leah J. Homad, Vanessa Rubin, Hayley Glantz, Moni Neradilek, Emilie Seydoux, Madeleine F. Jennewein, Anna J. MacCamy, Junli Feng, Gregory Mize, Stephen C. De Rosa, Andrés Finzi, Maria P. Lemos, Kristen W. Cohen, Zoe Moodie, M. Juliana McElrath, Andrew T. McGuire

**Affiliations:** 1 Vaccine and Infectious Disease Division, Fred Hutchinson Cancer Research Center, Seattle, WA, USA.; 2 Department of Global Health, University of Washington, Seattle, WA, USA.; 3 Department of Laboratory Medicine and Pathology, University of Washington, Seattle, WA, USA.; 4 Centre de Recherche du CHUM, Montréal, QC, Canada.; 5 Département de Microbiologie, Infectiologie et Immunologie, Université de Montréal, Montreal, QC, Canada.; 6 Department of Microbiology and Immunology, McGill University, Montreal, QC, Canada.; 7 Department of Medicine, University of Washington, Seattle, WA, USA.

## Abstract

Emerging severe acute respiratory syndrome coronavirus 2 (SARS-CoV-2) variants have raised concerns about resistance to neutralizing antibodies elicited by previous infection or vaccination. We examined whether sera from recovered and naïve donors, collected before and after immunizations with existing messenger RNA (mRNA) vaccines, could neutralize the Wuhan-Hu-1 and B.1.351 variants. Prevaccination sera from recovered donors neutralized Wuhan-Hu-1 and sporadically neutralized B.1.351, but a single immunization boosted neutralizing titers against all variants and SARS-CoV-1 by up to 1000-fold. Neutralization was a result of antibodies targeting the receptor binding domain and was not boosted by a second immunization. Immunization of naïve donors also elicited cross-neutralizing responses but at lower titers. Our study highlights the importance of vaccinating both uninfected and previously infected persons to elicit cross-variant neutralizing antibodies.

## Boosterism could save lives

Postinfection immune protection against severe acute respiratory syndrome coronavirus 2 reinfection is not fully understood. It will be devastating if waves of new variants emerge that undermine natural immune protection. Stamatatos *et al.* investigated immune responsiveness 4 to 8 months after previously infected individuals were given a messenger RNA–based vaccine developed for the original Wuhan variant (see the Perspective by Crotty). Before vaccination, postinfection serum antibody neutralization responses to virus variants were variable and weak. Vaccination elevated postinfection serum-neutralizing capacity approximately 1000-fold against Wuhan-Hu-1 and other strains, and serum neutralization against the variant B.1.351 was enhanced. Although responses were relatively muted against the variant, they still showed characteristic memory responses. Vaccination with the Wuhan-Hu-1 variant may thus offer a valuable boost to protective responses against subsequent infection with variant viruses.

*Science*, abg9175, this issue p. 1413; see also abj2258, p. 1392

## Teaser

Previous infection results in enhanced vaccine-induced immune protection against variant B.1.351 and other variants.

The severe acute respiratory syndrome coronavirus 2 (SARS-CoV-2) betacoronavirus first emerged in the Hubei Province of China in late 2019 and has since infected more than 115 million people and caused more than 2.5 million deaths in 192 countries (*1*–*3*). Infection is mediated by the viral spike protein (S), which is composed of an S1 domain that contains an N-terminal domain (NTD), a C-terminal domain (CTD), and a receptor binding domain (RBD) that mediates attachment to the entry receptor angiotensin-converting enzyme 2 (ACE2) as well as an S2 domain that contains the fusion machinery (*4*–*8*).

Preexisting immunity to SARS-CoV-2 is associated with protection against reinfection in humans (*9*–*11*) and in nonhuman primates (*12*, *13*). Although the correlates of protection in humans against repeat infection or after vaccination have not been firmly established, neutralizing antibodies (nAbs) are thought to be an important component of a protective immune response against SARS-CoV-2 (*14*, *15*). In support of this, passive transfer of nAbs limits respiratory tract infection and protects against infection in animal models (*16*–*20*), and nAbs may contribute to protection against infection in humans (*9*). SARS-CoV-2 infection rapidly elicits nAbs (*16*, *21*–*24*) that decline, but remain detectable, over several months (*25*–*29*).

Most serum nAb responses elicited during natural infection are directed at the RBD (*21*, *23*, *30*, *31*). Numerous neutralizing anti-RBD monoclonal antibodies (mAbs) have been characterized, the most potent of which block the RBD-ACE2 interaction (*16*, *17*, *22*–*24*, *32*–*37*). Neutralizing mAbs that bind regions of the viral spike have also been identified (*24*, *33*, *38*–*42*).

Two mRNA-based vaccines (Pfizer-BioNTech BNT162b2 and Moderna mRNA-1273) have received emergency use authorization in several countries. Both vaccines encode a stabilized ectodomain version of the S protein derived from the Wuhan-Hu-1 variant isolated in December 2019 (*43*), show >94% efficacy at preventing COVID-19 illness (*44*–*47*), and elicit nAbs (*48*, *49*).

Because of the high global burden of SARS-CoV-2 transmission, viral evolution is occurring. Recently, viral variants of concern have emerged in the UK (B.1.1.7), South Africa (B.1.351), and Brazil (P.1) that harbor specific mutations in their S proteins that may be associated with increased transmissibility (*50*–*55*).

Of particular concern are mutations found in the B.1.351 lineage, which is defined by the D80A (amino acid substitution from aspartic acid to alanine at position 80) and D215G mutations in the NTD; the K417N, E484K, and N501Y mutations in the RBD; and the D614G mutation in S1 (*52*, *56*). An A701V mutation in S2 is also observed at high frequencies, whereas deletions in residues 242 to 244 as well as R246I and L18F mutations in the NTD are present at lower frequencies (*52*). (Single-letter abbreviations for the amino acid residues are as follows: A, Ala; C, Cys; D, Asp; E, Glu; F, Phe; G, Gly; H, His; I, Ile; K, Lys; L, Leu; M, Met; N, Asn; P, Pro; Q, Gln; R, Arg; S, Ser; T, Thr; V, Val; W, Trp; and Y, Tyr.)

The B.1.1.7, B.1.351, and P.1 lineages all harbor a N501Y mutation in the RBD, which increases the affinity for the ACE2 receptor (*57*, *58*), and a D614G mutation, which increases virion spike density, infectivity, and transmissibility (*59*, *60*). The B.1.351 and P.1 lineages also share the E484K mutation in the RBD, and both variants are mutated at position 417 (K417T in P.1).

Mutations found in emergent S variants decrease sensitivity to neutralization by mAbs, convalescent plasma, and sera from vaccinated individuals (*27*, *37*, *58*, *61*–*70*). As a result, there is concern that these and other emerging variants can evade nAb responses generated during infection with variants that were circulating earlier in the pandemic and also nAb responses elicited by vaccines based on the S protein of the Wuhan-Hu-1 variant. There is concern that these mutations are responsible for the reduced efficacy observed in ongoing trials of SARS-CoV-2 vaccines in South Africa (*71*, *72*).

Here, we evaluated the neutralization susceptibility of spike variants harboring lineage-defining and prevalent B.1.351 mutations to sera from two groups. Sera were collected from 15 donors with previously confirmed SARS-CoV-2 infection [referred to as previously infected donors (PIDs)] before and after one or two immunizations with either mRNA vaccine and from 13 uninfected donors who received two doses of the above vaccines [referred to as naïve donors (NDs); tables S1 and S2].

Antibody neutralization experiments were performed with pseudoviruses expressing either the full-length Wuhan-Hu-1 S or either of two versions of the B.1.351 lineage S—one herein referred to as B.1.351, containing the lineage-defining S mutations D80A, D215G, K417N, E484K, N501Y, and D614G and the A701V mutation that is highly prevalent in this lineage, and a second variant that also includes a Δ242-243 deletion (B.1.351–Δ242-243). The viral stocks were appropriately diluted to achieve comparable entry levels during the neutralization experiments (fig. S1).

**Fig. 1:**
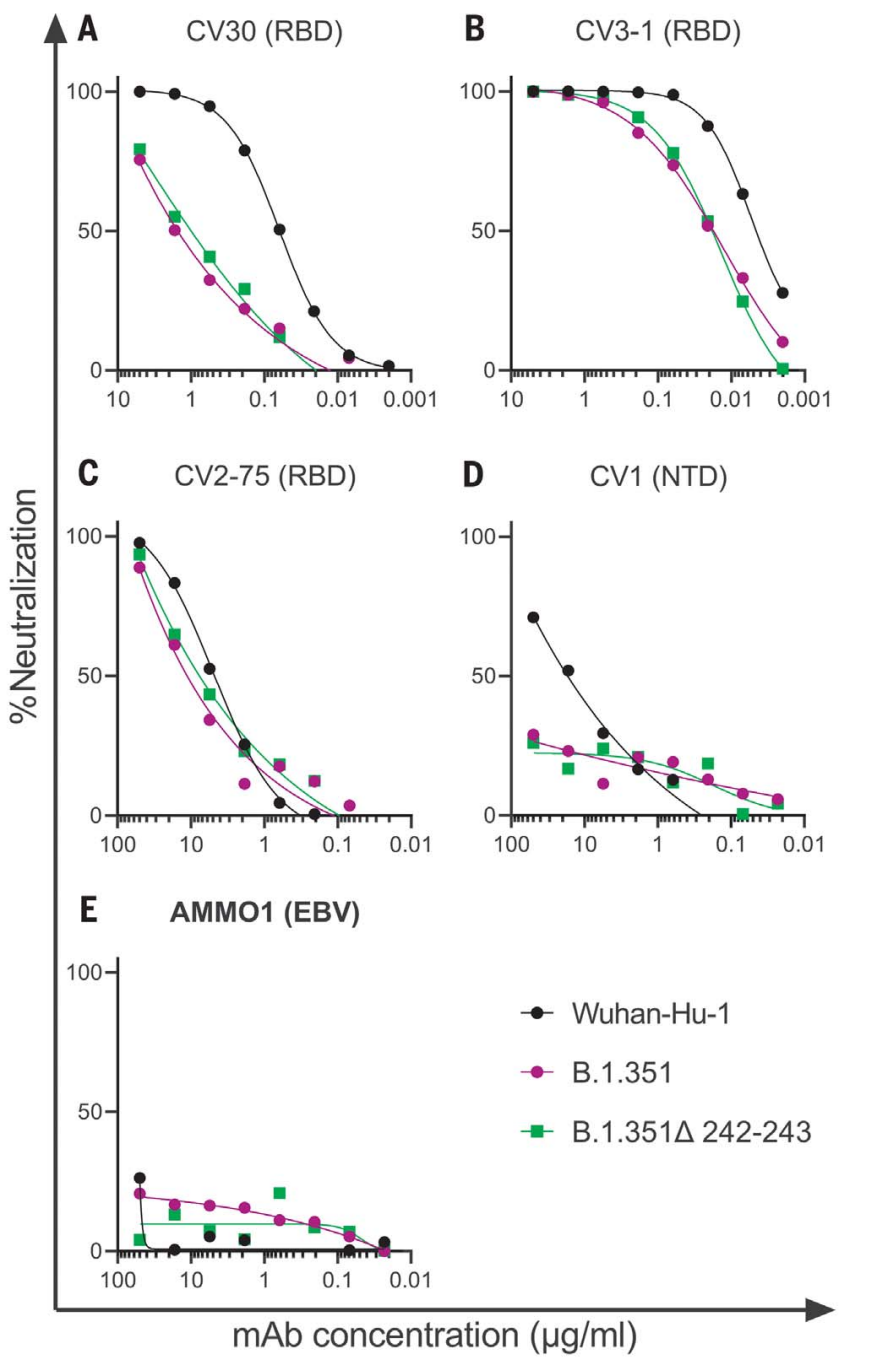
B.1.351 variants show decreased susceptibility to neutralizing mAbs. (**A** to **E**) The ability of the indicated mAbs to neutralize Wuhan-Hu-1, B.1.351, and B.1.351–Δ242-243 pseudovirus infectivity in 293T-hACE2 cells was measured as indicated. The epitope specificity of each mAb is shown in parentheses. EBV, Epstein-Barr virus. Data points represent the mean of two technical replicates. Data are representative of two independent experiments.

We first evaluated the neutralizing potency of several mAbs isolated from nonvaccinated patients infected early in the pandemic. These mAbs target different epitopes: three against the RBD (CV30, CV3-1, and CV2-75) and one against the NTD (CV1) (fig. S2). CV30 is a member of the VH3-53 class of antibodies that bind to the receptor binding motif (RBM) (*22*, *32*, *73*–*78*). It makes direct contact with the K417 and N501 residues in the RBM that are mutated in the B.1.351 and P.1 lineages; however, unlike other known VH3-53 mAbs, it does not contact E484 (*78*). The neutralization potency of this mAb was ~10-fold weaker toward both B.1.351 variants (Fig. 1A). Similarly, the non–VH3-53 mAb CV3-1 was three- to fourfold less potent against the B.1.351 variants (Fig. 1B), whereas CV2-75 was modestly less effective (Fig. 1C). By contrast, the anti-NTD CV1 mAb was unable to neutralize either B.1.351 variant (Fig. 1D). As expected, the control anti–Epstein-Barr virus mAb AMMO1 was nonneutralizing (*79*) (Fig. 1E). Collectively, these data indicate that the B.1.351 variants tested here are more resistant to neutralization by mAbs isolated from subjects infected by viral variants from early in the pandemic. We therefore examined whether the B.1.351 variants are resistant to nAb responses elicited by the Pfizer-BioNTech or Moderna mRNA vaccines in both PIDs and NDs.

**Fig. 2:**
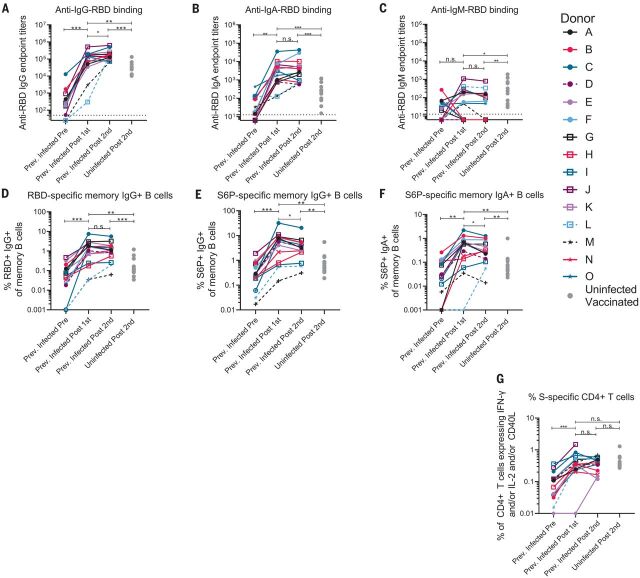
A single dose of a spike-derived mRNA vaccine elicits a strong recall response. (**A** to **C**) IgG (A), IgA (B), and IgM (C) end-point antibody titers specific to the RBD of the Wuhan-Hu-1 variant were measured in serum collected from PIDs before and after one or two immunizations with the Pfizer-BioNTech or Moderna mRNA vaccines by ELISA, as indicated. End-point titers measured in sera from NDs after two vaccine doses are shown for comparison (gray dots). (**D**) Frequency of Wuhan-Hu-1 RBD-specific IgG^+^ memory B cells (live, IgD^−^, CD19^+^, CD20^+^, CD3^−^, CD14, CD56^−^, singlet, and lymphocytes) in peripheral blood mononuclear cells (PBMCs) from PIDs was measured before and after one or two immunizations. (**E** and **F**) The frequency of S6P-specific IgG^+^ (E) and IgA^+^ (F) memory B cells in PBMCs from PIDs was measured before and after one or two immunizations. The frequencies of memory B cells from NDs after two vaccine doses are shown for comparison in (D) to (F) (gray dots). (**G**) The frequency of S-specific CD4^+^ T cells expressing interferon-γ (IFN-γ) and/or interleukin-2 (IL-2) and/or CD40L in PBMCs from PIDs was measured before and after one or two immunizations. The frequencies of S-specific CD4^+^ T cells in PBMCs from uninfected donors after two vaccine doses are shown for comparison (gray dots). Experiments were performed once. Significant differences in infected donors before or after vaccination [(A) to (G)] were determined using a Wilcoxon signed rank test (n.s., not significant; *P < 0.05; **P < 0.01; and ***P < 0.001). Significant differences between previously infected and uninfected donors [(A) to (G)] were determined using a Wilcoxon rank sum test (*P < 0.05; **P < 0.01; and ***P < 0.001).

The RBD-specific immunoglobulin G (IgG), IgM, and IgA binding responses to the RBD from the Wuhan-Hu-1 variant were measured before (on average, 202 days after symptom onset; table S1) and either 5 to 29 days (table S1) after the first and second immunizations in the PIDs or 6 to 28 days after the second immunization in the NDs. Three PIDs experienced asymptomatic SARS-CoV-2 infection (donors D, L, and M; table S1), two of whom, L and M, did not have detectable anti-RBD IgG antibodies before immunization, whereas the third, D, had low but detectable serum anti-RBD IgG antibody titers (Fig. 2A). In the 13 PIDs with RBD-specific IgG antibodies before vaccination, a single dose of either vaccine boosted these titers ~500-fold (Fig. 2A). Across all PIDs, there was a 200-fold increase in median RBD-specific IgA titers after vaccination (Fig. 2B). Overall, in PIDs, a single vaccine dose elicited 4.5-fold higher IgG and 7.7-fold higher IgA titers compared with two vaccinations in NDs. RBD-specific IgM titers were generally lower and were not significantly boosted in response to vaccination in PIDs (Fig. 2C). In PIDs, a concomitant increase in RBD- (Fig. 2D) and S-specific IgG^+^ (Fig. 2E) memory B cell frequencies took place after vaccination. The two PIDs that lacked RBD-specific IgG titers before immunization (donors L and M) also lacked RBD-specific IgG^+^ memory B cells (Fig. 2D) and had lower frequencies of S-specific IgG^+^ memory B cells after vaccination. Consistent with the serology data, an increase in the frequency of IgA^+^ (Fig. 2F) but not IgM^+^ spike-specific memory B cells was observed (fig. S3). Vaccination also induced S-specific CD4^+^ T cell responses (Fig. 2G).

**Fig. 3:**
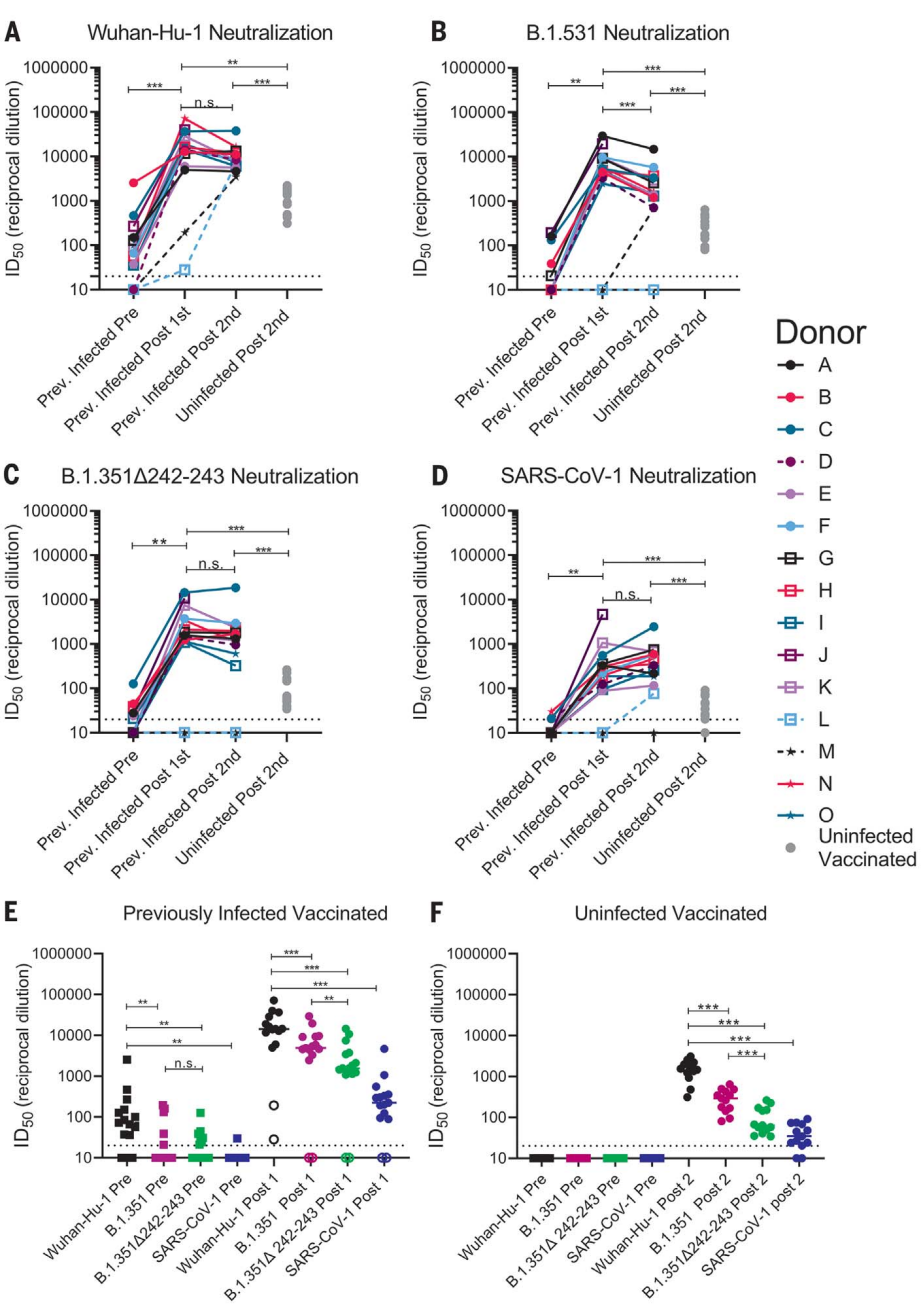
Preexisting SARS-CoV-2 nAb responses are boosted by a single dose of a spike-derived mRNA vaccine. (**A** to **D**) The serum dilution resulting in 50% neutralization (ID_50_) of Wuhan-Hu-1 (A), B.1.351 (B), B.1.351–Δ242-243 (C), and SARS-CoV-1 (D) pseudoviruses was measured in PIDs before and after one or two immunizations with the Pfizer-BioNTech or Moderna vaccines and in NDs after two vaccine doses, as indicated. Data points between PIDs who were symptomatic and asymptomatic are connected by solid and dashed lines, respectively, in (A) to (D). (**E**) Serum dilution resulting in 50% neutralization (ID_50_) from PIDs before (squares) and after (circles) a single immunization with the Pfizer-BioNTech or Moderna vaccines against Wuhan-Hu-1, B.1.351, B.1.351–Δ242-243, and SARS-CoV-1 pseudoviruses, as indicated. PIDs who were asymptomatic and negative for anti-IgG RBD antibodies and RBD-specific IgG^+^ memory B cells before vaccination are shown as open circles. (**F**) Neutralizing potency (ID_50_) of serum from NDs after two immunizations with the Pfizer-BioNTech or Moderna vaccines against the indicated pseudoviruses. Each data point represents a different donor, and the horizonal bars represent the medians in (E) and (F). The dashed lines demarcate the lowest serum dilutions tested. Experiments were performed once. Significant differences in infected donors before or after vaccination, or from the same time point against different variants, were determined using a Wilcoxon signed rank test (*P < 0.05; **P < 0.01; and ***P < 0.001). Significant differences between previously infected and uninfected donors were determined using a Wilcoxon rank sum test (*P < 0.05; **P < 0.01; and ***P < 0.001).

Sera from 12 of 15 PIDs sampled before vaccination neutralized the Wuhan-Hu-1 SARS-CoV-2 variant (Fig. 3A and fig. S4). The nonneutralizing sera were from the three asymptomatic PIDs who had low or undetectable anti-RBD IgG titers (Fig. 3A, dashed lines, and fig. S4). Prevaccine sera from the NDs were also nonneutralizing (fig. S5). Consistent with the observed increase in binding antibodies after a single immunization in PIDs with preexisting RBD-specific IgG titers, the median half-maximal neutralizing titers [half-maximal inhibitory dilution (ID_50_)] were boosted ~1000-fold after the first dose, whereas the second dose had no effect (Fig. 3A). In the two PIDs lacking RBD-specific IgG titers before vaccination, the first vaccine dose elicited lower neutralizing titers (ID_50_ = ~30 in donor L and ~200 in donor M; Fig. 3A). In the NDs, two doses of the vaccine elicited ID_50_ titers that were ~10- and 5-fold lower than those elicited by one or two doses in the PIDs, respectively (Fig. 3A and fig. S6). Collectively, these data indicate that in PIDs who generate adequate immunological memory to the RBD, a single vaccine dose elicits an anamnestic response resulting in RBD-binding and nAb responses that are superior to a two-dose regimen in uninfected donors. A similar boost in binding and/or vaccine-matched neutralizing titers has been observed in PIDs who received a single mRNA vaccine dose in two recent studies (*80*, *81*).

We next evaluated the ability of sera collected before and after immunization in NDs and PIDs to neutralize the more resistant B.1.351 and B.1.351–Δ242-243 pseudoviruses. These variants are 0.5 and 0.7% divergent from the Wuhan-Hu-1 variant. We also included SARS-CoV-1 pseudoviruses in this analysis as a representative variant that is even more dissimilar to the vaccine. SARS-CoV-1 and SARS-CoV-2 are 24, 26, and 50% divergent in the overall S protein, RBD, and RBM, respectively (*82*). Consequently, several mAbs that potently neutralize SARS-CoV-2 fail to bind SARS-CoV-1 (*16*, *22*–*24*).

Before vaccination, 5 of 15 sera from PIDs neutralized B.1.351, and only three had ID_50_ titers above 100 (Fig. 3, B and E, and fig. S4); 7 of 15 neutralized B.1.351–Δ242-243, and only one had titers above 100 (Fig. 3, C and E, and fig. S4). Only two prevaccine PID sera achieved 80% neutralization of B.1.351, and only one achieved 80% neutralization of B.1.351–Δ242-243 (fig. S7A). The median ID_50_ of the prevaccine sera against the Wuhan-Hu-1 variant was significantly higher than that against B.1.351 or B.1.351–Δ242-243 (Fig. 3E). Consistent with the high level of sequence disparity, sera from only one PID showed very weak neutralizing activity toward SARS-CoV-1 before vaccination (Fig. 3, D and E, and fig. S7).

A single immunization boosted the nAb titers against all three SARS-CoV-2 variants and SARS-CoV-1 in 13 of 15 PIDs (Fig. 3, A to D); however, the median ID_50_ titers were ~3-fold lower against B.1.351, ~10-fold lower against B.1.351–Δ242-243, and 100-fold lower against SARS-CoV-1 than against Wuhan-Hu-1 (Fig. 3E). A single immunization did not elicit nAbs against the B.1.351 variants or SARS-CoV-1 in the two asymptomatic donors who lacked RBD-specific IgG memory (donor L and M; Fig. 3, A to D, and Fig. 3E, open circles). The median ID_80_ values were also lower for the B.1.351 and B.1.351–Δ242-243 variants compared with the Wuhan-Hu-1 variant (fig. S7A).

The neutralizing titers elicited by a single immunization in PIDs were significantly higher than those elicited by two immunizations in NDs against all pseudoviruses tested—10-fold higher against Wuhan-Hu-1 (Fig. 3A), 20-fold higher against B.1.351 (Fig. 3B), 30-fold higher against B.1.351–Δ242-243 (Fig. 3C), and 7-fold higher against SARS-CoV-1 (Fig. 3D). Only 8 of 13 vaccinated NDs were able to achieve 80% neutralization of B.1.351–Δ242-243, and none could achieve 80% neutralization of SARS-CoV-1 (fig. S7B).

**Fig. 4:**
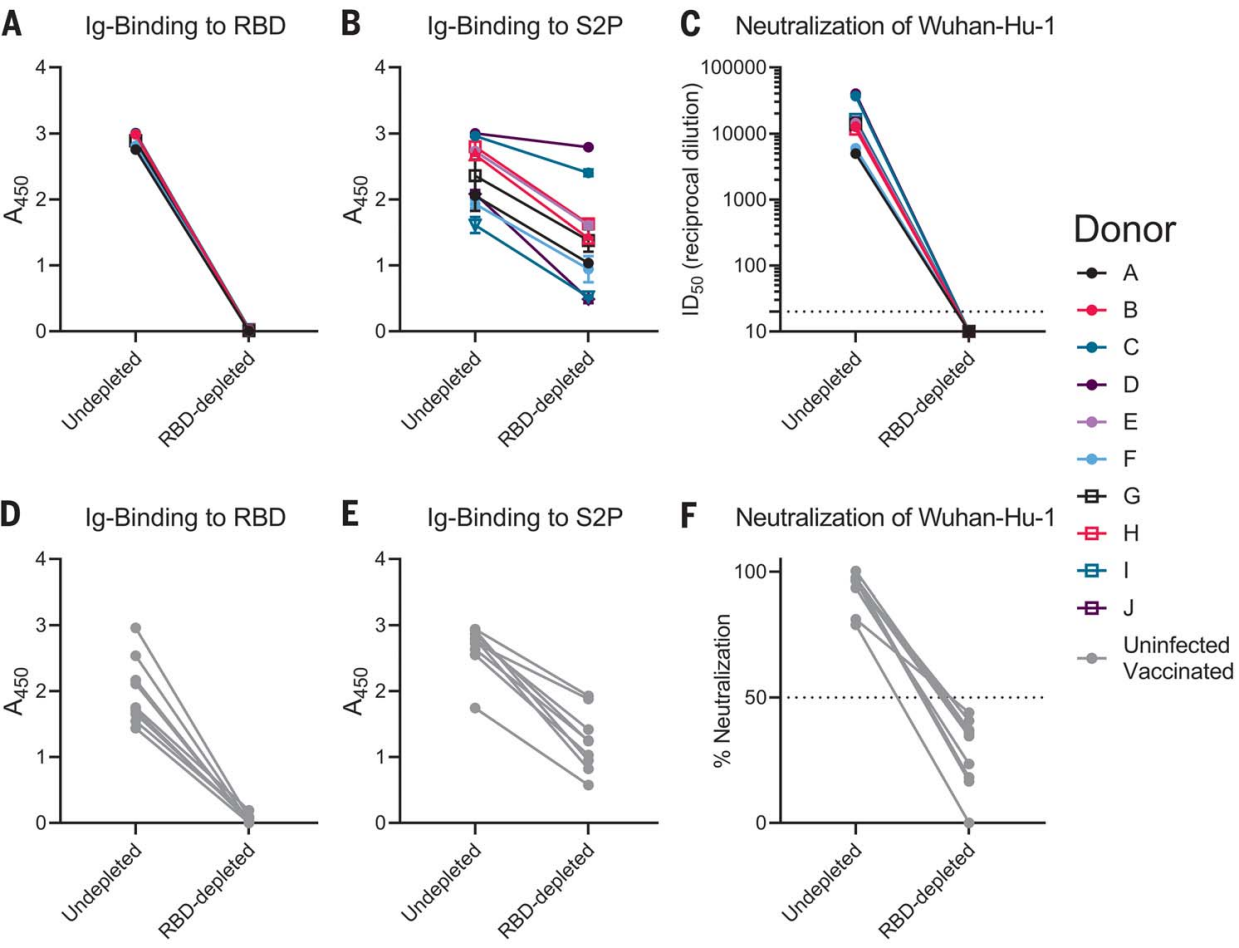
Vaccine-elicited nAbs target the RBD. RBD-binding antibodies were adsorbed from sera from PIDs after receiving a single vaccine dose or from NDs after receiving two vaccine doses using Wuhan-Hu-1 RBD immobilized to magnetic beads. (**A** and **B**) Antibody binding in undepleted or RBD-depleted sera from PIDs was measured to RBD at a 1:500 dilution (A) and S2P at a 1:4500 dilution (B) by ELISA, as indicated. A_450_, absorbance at 450 nM. (**C**) The serum dilution resulting in 50% neutralization (ID_50_) of the Wuhan-Hu-1 pseudovirus was measured in undepleted or RBD-depleted sera from the PIDs in (A) and (B). (**D** and **E**) Antibody binding in undepleted and RBD-depleted sera from NDs was measured to RBD at a 1:500 dilution (D) and S2P at a 1:500 dilution (E) by ELISA. (**F**) The percent neutralization of a 1:120 dilution of undepleted or RBD-depleted sera from the donors in (D) and (E) was measured against the Wuhan-Hu-1 pseudovirus. Experiments were performed once.

The B.1.351 and B.1.351–Δ242-243 variants contain three RBD mutations that affect the neutralization potency of anti-RBD mAbs (Fig. 1). Moreover, preexisting anti-RBD IgG memory appears to be important for a robust recall response to vaccination. To determine the relative contribution of anti-RBD antibodies to serum neutralization, we depleted RBD-specific antibodies from the sera of 10 PIDs after one vaccination and from nine NDs after two vaccinations. This approach efficiently removed RBD-specific (Fig. 4, A and C) but not anti-S2P–specific antibodies from sera, as measured by enzyme-linked immunosorbent assay (ELISA) (Fig. 4, B and D). This depletion abrogated serum neutralization of Wuhan-Hu-1 virus (Fig. 4, C and F), which suggests that most nAbs elicited or boosted by vaccination target this subdomain.

The above results indicate that in NDs, two doses of either the Pfizer-BioNTech or Moderna vaccines elicited nAb titers against the vaccine-matched Wuhan-Hu-1, lower titers against B.1.351, and even lower titers against B.1.351–Δ242-243. Reduced sensitivity to vaccine-elicited nAbs has been reported for other B.1.351 variants (*66*, *83*, *84*).

Similarly, sera from PIDs who experienced symptomatic SARS-CoV-2 infection and who had detectable anti-RBD IgG titers before vaccination displayed generally weak nAb titers against Wuhan-Hu-1 at 1 to 9 months after infection and lower or nonexistent titers against the B.1.351 variants, in agreement with another study (*69*). However, as long as RBD-specific IgG^+^ memory B cell and antibody responses were generated during infection, a single immunization with either mRNA vaccine elicited a robust recall response that boosted the autologous neutralizing titers by ~1000-fold, and these antibody responses cross-neutralized the B.1.351 variants, but at lower titers. In most of the previously infected vaccinees, the anti–B.1.351–Δ242-243 neutralizing titers were comparable to those against the vaccine-matched Wuhan-Hu-1 in uninfected vaccinees. This is notable, as these titers were associated with 95% protection from COVID-19 in phase 3 trials (*44*, *46*, *48*, *49*). Moreover, vaccine-elicited antibody responses also neutralized SARS-CoV-1 but with much lower potencies. Collectively, our data suggest that the two mRNA vaccines that are based on the Wuhan-Hu-1 variant can elicit and/or boost nAb responses but that their potency is reduced against divergent variants.

Here, we show that the cross-nAb responses generated after immunization in previously infected subjects are a result of anti-RBD antibodies. Combined with the observation that the vaccines elicited nAb responses that are less potent against the B.1.351 variant with the Δ242-243 deletion in the NTD, this suggests that NTD mutations can modulate the sensitivity of emerging variants to anti-RBD nAbs. By contrast, the NTD region itself, which appears to tolerate antigenic variation in SARS-CoV-2 and other coronaviruses (*50*, *52*, *55*, *85*), does not appear to be the target of cross-nAbs elicited by infection or vaccination. We note that there are other less-frequent mutations associated with this lineage, such as L18F, Δ244, L244H, and R246I, that were not examined here, which may further increase resistance to vaccine-elicited antibodies. In this study, a pseudovirus assay was used to measure nAbs. Several studies have now shown that authentic virus and pseudovirus neutralization correlate quite well (*16*, *86*, *87*). Although the absolute sensitivity of the authentic and pseudovirus assays may differ, we anticipate that the relative differences we report here will not vary between the two.

Although the correlates of protection for SARS-CoV-2 vaccines have not been established, studies in nonhuman primates indicate that even low titers of nAbs are sufficient to prevent experimental SARS-CoV-2 infection, particularly if CD8^+^ T cell responses are mounted (*18*). Our study suggests that most previously infected subjects will benefit from a single immunization with either the Pfizer-BioNTech or Moderna vaccines, as it will lead to significant increases in serum nAb responses against vaccine-matched and emerging variants. The observation that a second dose administered 3 to 4 weeks after the first did not further boost neutralizing titers in PIDs who have clear evidence of RBD-directed immunological memory before vaccination suggests that the second dose of an mRNA vaccine could be delayed in some persons who have previously been infected with SARS-CoV-2. Longitudinal monitoring of the nAb titers before and after the first dose should be used to determine the necessity or optimal timing of the second dose in the context of previous infection.

## Supplementary Materials

science.sciencemag.org/content/372/6549/1413/suppl/DC1
Materials and Methods Figs. S1 to S8 Tables S1 to S4 References (*89*–*95*) MDAR Reproducibility Checklist

## Appendix

View/request a protocol for this paper from *Bio-protocol*.
